# The Role of Psychological Factors in Older Adults’ Readiness to Use eHealth Technology: Cross-Sectional Questionnaire Study

**DOI:** 10.2196/14670

**Published:** 2020-05-28

**Authors:** Lenka Knapova, Adam Klocek, Steriani Elavsky

**Affiliations:** 1 Institute for Research on Children, Youth and Family Masaryk University Brno Czech Republic; 2 Faculty of Informatics Masaryk University Brno Czech Republic; 3 Faculty of Education University of Ostrava Ostrava Czech Republic; 4 Faculty of Social Studies Masaryk University Brno Czech Republic

**Keywords:** eHealth, information technology, need for cognitive closure, elderly

## Abstract

**Background:**

Information and communication technology (ICT) use among older adults has been on the rise in recent years. However, the predictors and mechanisms behind older adults’ acceptance and use of ICT are not clear.

**Objective:**

This study aimed to systematically describe ICT usage among Czech older adults and to evaluate the factors influencing their ICT use and readiness to use digital technology to promote health (eHealth readiness). The primary focus was on psychological factors and the role of persons close to older adults.

**Methods:**

The research utilized cross-sectional survey data from a quota-based sample of Czech older adults (>50 years) and persons close to them further referred to as *close persons* (N=250 dyads). A structural equation modeling framework was used to evaluate relationships between psychological factors, ICT use, and eHealth readiness.

**Results:**

Czech older adults’ use of ICT is low with the exception of cell phone usage (cell phone usage by 173/250, 69.2%; other devices used by 50/250, 20.0% of older adults or less). Apart from age (β=−.21; *P*<.001), eHealth readiness was predicted by ICT use (β=.65; *P*<.001). eHealth readiness was also indirectly affected by the need for cognitive closure (NFCC): individuals with a high need for closure perceived more barriers to ICT (β=.23; *P*=.01) and more reported barriers were linked to lower ICT usage (β=−.21; *P*=.001). The expected positive relationships between eHealth readiness of persons close to older adults and ICT use and eHealth readiness of older adults were not significant, but the total effect of eHealth readiness of persons close to older adults on eHealth readiness of older adults was positive and significant (β=.18; *P*=.01), indicating some level of influence of persons close to them on older adults’ attitudes and behaviors.

**Conclusions:**

This study provided the first systematic examination of Czech older adults’ ICT usage and eHealth readiness. Novel predictors (NFCC and close persons’ variables) were evaluated and yielded actionable results. More research is needed to clarify the role of persons close to older adults.

## Introduction

### Background

Older adults (>65 years) are the fastest-growing segment of the population, estimated to account for more than 25% of the total population by 2050 and outnumbering the youngest segment of children under the age of 15 years by 2045 [1,2]. In sharp contrast with the youngsters, older adults are usually portrayed as uninterested in the ever-evolving technological advancements, and thus, lacking familiarity with information and communication technologies (ICTs). However, recent data indicate a sharp increase in technology use among older adults globally, and the Czech Republic has been no exception to this trend. Whereas in 2012, 79.4%, 56.2%, and 17.3% of Czech adults aged 45 to 54 years, 55 to 64 years, and older than 65 years, respectively, used a computer, these numbers have increased to 88.8%, 73.3%, and 32.8% for the respective age groups in 2017 [3]. A similar increase can be seen in the United States, where the percentage of smartphone users older than 65 years rose from 18% in 2013 to 42% in 2017 [4]. Data refined by age and economic activity further support the trend of seniors as the fastest-growing segment of ICT users. The subgroup with the largest increase in the percentage of computer users among Czechs are pensioners, particularly the retired, with only 20.9% of computer users in 2012 compared with 36.1% in 2017 [3].

Considering the position of the Czech Republic within the European Union (EU), rates of ICT usage are slightly below the average for member countries of the EU. For instance, the percentage of households connected to the internet in the Czech Republic is 81.7% as compared with the EU average of 85.4%. The disparities are much more evident in the older segment of the population and for new technological developments such as smartphones and mobile internet. Only 13.1% of Czech adults aged between 55 and 74 years use mobile phones to connect to the internet, which stands in sharp contrast to the average of 34.2% for EU member countries. Within the EU, the Netherlands, Denmark, and Luxembourg rank the highest in terms of older adults using mobile internet, with 61.8%, 61.4%, and 61.1%, respectively. Curiously, the Czech Republic lags behind even when compared with similar countries (by gross domestic product per capita) such as Hungary, Croatia, and Slovakia with 25.2%, 24.2%, and 18.6% of older mobile internet users, respectively [3]. The lower adoption rates of new ICT technologies (eg, mobile internet) among Czech older adults beg the question of how ready Czech older adults are for the deployment of novel ICT approaches in the field of health and medical services, a notion that has been supported by institutions such as the US Department of Health and Human Services or the European Commission and is incorporated within the EU’s strategic goals (eg, Europe 2020 strategy) [5]. If left to persist or widen, the digital divide could exacerbate isolation of older adults and may further disadvantage older adults when seeking services that are rapidly being transformed into the digital domain (eg, mobile banking and Web-based reservation systems) or prevent them from adopting newer technologies (eg, assistive technologies) [6,7].

### Information and Communication Technology for Health

The use of ICT has been argued to have the potential to positively influence older adults’ well-being, decrease loneliness, and increase social support and integration into society [8-10]. This could be especially true with new emerging internet-connected or mobile technologies aimed specifically at improving health and health-related behaviors. Specifically, the term electronic health (eHealth) is used to describe healthy behavior promoting interventions and devices which make use of the internet (eg, computers, personal digital assistants, cell phones, smartphones). Mobile health (mHealth) denotes the use of mobile handheld devices in support of medical and public health practice [11]. The use of technologies such as smartphones, smartphone-based apps, and integrated or connected sensors provides new ways to monitor and improve one’s health, healthy lifestyle, and overall well-being. To the point, recent studies support the effectiveness of such mHealth or eHealth interventions for improving health-related behaviors, for example, increased physical activity, sleep, or reduced sedentary behavior [12,13]. For example, Muellman et al [12] conducted a systematic review and found that eHealth interventions promoting physical activity delivered through computer or handheld devices led to increased levels of physical activity in adults aged 55 years and older. Similarly, in a recent review, Elavsky et al [13] concluded that there is evidence supporting the effectiveness of mHealth interventions (defined as treatment programs delivered at least partially by a mobile phone, a smartphone, or a tablet) for increasing physical activity and reducing sedentary behavior in adults aged 50 years and older.

Nevertheless, a vital premise for the effectiveness of eHealth interventions is the initial acceptance of the intervention or program as well as adherence to it over time. Uptake of health-promoting technologies is very low even in carefully conducted research conditions [14], and the continued use of the eHealth technology significantly decreases over time [15]. One factor that can contribute both to the low adherence rates observed in existing interventions as well as the slow uptake of eHealth technologies may be low eHealth readiness. Evaluating how prepared individuals are to adopt eHealth technology or intervention might be exceptionally beneficial when studying older adults whose current adoption and usage of eHealth technologies might be low.

### Factors Influencing Information and Communication Technology Acceptance

From a socio-ecological perspective, ICT acceptance among older adults could be best explained by the interaction of factors at multiple levels. At the individual level, ICT use and adoption of new technological developments has been consistently associated with sociodemographic characteristics including age [16-19], education [17-20], income or socioeconomic status [18,21], and marital status [17,18,20]. Vroman et al [17] summarize that nonusers tend to be 75 years or older, have a disability or a chronic health problem, live alone, be single or widowed, and have a lower level of education than ICT users.

Among psychological characteristics, explanations of technology use focus on individual differences in motivations to use technology (eg, technology acceptance model and its variations or hedonic motivation) [22,23], attitudinal factors (eg, perceived ease or usefulness) [24], self-efficacy toward the system [25,26], or anxiety toward the system [26]. In addition, better cognitive abilities, including memory, learning, and concentration, have been linked to higher technology usage [18,27-29]. Dispositional personality characteristics have been acknowledged as potential drivers of ICT adoption and use among older adults as well [17,18]. However, few studies have been conducted so far. One recent study evaluated 17 individual difference predictors of ICT use, indexed with a checklist of 10 different ICTs, including ICTs for health [30]. Need for cognition, defined as an individual’s tendency to engage in and enjoy activities that require thinking [31], perceived mastery, and optimism, was found to positively predict ICT use, whereas cynical hostility emerged as a negative predictor [30]. Need for cognition also positively influenced the perceived benefits of ICT use and negatively influenced perceived barriers of ICT use. However, this study was exploratory and included a large number of hypotheses evaluated on one sample without performing any correction for multiple comparisons. Another personality trait which has not been widely studied in the context of ICT use and adoption but might have important implications is the need for cognitive closure (NFCC). NFCC is defined as a desire for a definite answer and an aversion toward ambiguity [32]. Interestingly, NFCC represents both a dimension of stable individual differences and a situationally evocable state [33]. Individuals high in dispositional NFCC feel a desire to quickly reach firm decisions; they are reluctant to have their decisions and views challenged, and they are resistant to information inconsistent with their views [33]. High NFCC individuals were found to be reluctant to change [34] and to be less willing to use innovative technology [35]. Individuals high in NFCC could thus exhibit more negative attitudes toward ICT, including new and innovative technology for improving health, be less prepared to use it, and show lower adoption levels.

Going beyond individual characteristics, other factors in older adults’ environments that have been found to affect technology usage of older adults include accessibility, financial support, hardware/software capacity and compatibility, and importantly also support, training, and assistance from others [18,36,37]. Vroman et al [17] add that older adults might be introduced to using ICT for its utility through natural exchanges that occur between family and friends (eg, by receiving hyperlinks via email to view family photos, products, or holiday destinations) and further use ICT as a utility (to search for health-related information, product, and services) and possibly also connect with a virtual community outside one’s geographical location. Indeed, previous studies have found that the involvement of family members, especially older adults’ children, positively influences ICT adoption [38]. More than 25% of Czech older adults reported that it was their children, grandchildren, or friends who brought them to use the internet [39]. In a similar vein, Vroman et al [17] found that older adults who live alone, are potentially isolated, and lack a social network are the least likely to use ICT.

### This Research

The objective of this study was to examine ICT use and eHealth readiness of Czech older adults and to evaluate the influence of psychological factors on older adults’ readiness to use eHealth technology, while considering the role of older adults’ close persons (eg, children and friends). It was hypothesized that older adults with a high NFCC would perceive more barriers to using ICT, use it less, and exhibit lower eHealth readiness. It was also hypothesized that close persons’ eHealth readiness would be positively related to older adults’ ICT use and eHealth readiness. Finally, as ICT use has been consistently demonstrated to decline with age and age has been shown to be an important predictor of ICT-related attitudes and behavior, the explanatory models tested considered the effect of age when evaluating the associations among NFCC, perceived barriers, current ICT use, and eHealth readiness.

## Methods

### Sample and Data Collection

The study was approved by the University Ethical Committee. A total of 250 Czech older adults and the persons close to them (close persons, N=250 dyads) participated in this research. The data were collected between September and November 2017. Participants were recruited and surveyed through a professional marketing and social research agency using stratified quota sampling. The quotas were set based on most recent census data (Czech Statistical Office) to correspond with the underlying population of adults aged 50 years and older based on region (representation of all 14 regions within the Czech republic with a quota based on resident population within each region), gender, age (50-59 years, 60-69 years, 70 years and older), education, and city size (categorized by number of inhabitants). The resulting primary sample of older adults is thus representative of the overall Czech population of adults of 50 years and older in terms of distribution by age, gender, education, region, and city size. Professional interviewers located in various regions were given quota breakdowns and conducted in-person questionnaires with corresponding participants until the quota was met. Each older respondent from the primary sample identified a close person (such as an adult child, a partner, or a friend) who at least occasionally helps them with day-to-day activities (eg, shopping, doctor’s visits, household chores, or running errands) with whom they are in contact at least once a week. All close persons were subsequently interviewed either in person or by telephone. The data from the primary sample of older adults were collected through standardized, structured face-to-face interviews (approximately 45 min in length) in the households of respondents with the help of a tablet and a questionnaire software. The data were collected at one time point and are cross-sectional.

### Measures

#### Demographics

Basic demographic information was collected (ie, age, gender, education, income, residence).

#### Information and Communication Technology Use

Participants provided information about the following devices: computer, laptop, cell phone, smartphone, and tablet. Participants were first asked to indicate the devices they own and use and subsequently report further details such as the daily usage time (hours/day) and length of use. This information was aggregated to form two measures of ICT use: *number of devices used* and *total usage time*. The *number of devices used* was calculated as a sum of all the devices participants reported to use (not own). The *total usage time* of the ICT devices per day was calculated as the sum of usage times per day provided for the specific devices. When a participant did not report usage of a device (and thus was not asked about the details of its use), the usage time was coded as 0. Responses for total reported usage time exceeding 24 hours per day (3 older adults, 12 close persons) were recoded to a maximum value of 16 hours per day to account for typical sleep duration and basic needs.

In addition, the use of the internet as a specific ICT-related technology was assessed. Participants were asked if they use the internet. Internet users were asked for further information about their *internet use,* such as the daily usage time (hours/day), length of use, and frequency of use. For nonusers of the internet, the usage time was coded as 0.

#### Perceived Barriers to Information and Communication Technology Use

Participants chose from the following list of barriers generated from previous studies on the topic: *Not enough knowledge*; *Technologies evolve too fast; I have trouble with vision and psychomotorics; I am afraid of making a mistake; I do not have enough support; Too expensive, I cannot afford it; I don’t care about internet; Something else.* Participants could indicate multiple barriers with the total number of perceived barriers ranging from 0 to 7.

#### Electronic Health Readiness

Both older adults and their close persons completed the eHealth Readiness scale by Bhalla et al [40]. The scale was translated to Czech using the method of double-back translation, with emphasis on systematic equivalence [41]. Inconsistencies in item wording were resolved by group discussion of study authors, which included senior researchers on the topic and bilingual speakers. The items were scored on a 6-point scale ranging from 1 (*completely disagree*) to 6 (*completely agree*). The total score was calculated as the sum of all the items. Bhalla et al [40] reported a Cronbach alpha of .81 and .83 for validation samples. Internal consistency in this research was higher: Cronbach alpha was .91 and .87 for the older adults and their close persons, respectively.

#### Need for Cognitive Closure

NFCC was measured with the Czech version of the 15-item NFCC Scale [42,43]. The items were scored on a 5-point scale ranging from 1 (*completely disagree*) to 5 (*completely* *agree*). The total score was calculated as the arithmetical mean of all the items. Širůček et al [43] reported a Cronbach alpha of .84. The Cronbach alpha in this research was .87.

### Data Analysis

Statistical analyses were performed using R (R Foundation for Statistical Computing), version 3.4.2 [44], packages psych (version 1.8.3.3), lavaan (version 0.5-23.1097), semPlot (version 1.1), and semTools (version 0.4-14).

The proposed relationships were tested using structural equation modeling (SEM). The structural model was estimated using the maximum likelihood with robust SEs test statistics estimator. The model fit was evaluated using standard measures of model fit: the standardized root mean square residual (SRMR), which should be less than or approximately 0.08 [45,46]; the root mean square error of approximation (RMSEA), with values below 0.05 indicating a close fit and values below 0.08 being less indicative of a good fit [47,48]; the comparative fit index (CFI), which should be higher than 0.90 [49]; and the Tucker-Lewis index (TLI) with recommended values greater than 0.90 [50] or 0.95 [46].

### Model Testing

First, we defined three latent variables. The latent variable of ICT use was specified as the combination of three manifest variables: number of used devices, total usage time of the devices, and internet usage time. The measurement model for eHealth readiness was a simple 1-factor model where all items were loaded on a single latent variable. eHealth readiness item 2 was excluded from the scale in both samples as it exhibited high residual correlations with other variables and worsened the overall fit of both the measurement model and the resulting structural model. Moreover, upon closer examination, the item (*I feel that my previous experience with online technologies is important to my success with using a lifestyle intervention*) intertwines previous experience with opinions about its importance, and the item wording makes it difficult to decide if an individual who has previous experience with online technologies but does not think this experience is important to one’s success should be labeled as more or less ready to use eHealth technology. For NFCC, a 1-factor model was defined where all NFCC items loaded on a single latent variable and error correlations between items from the same facet were allowed to account for their common facet source (ie, residual correlations were allowed for triads of items 1-3, 4-6, 7-9, 10-12, and 13-15).

**Figure 1:**
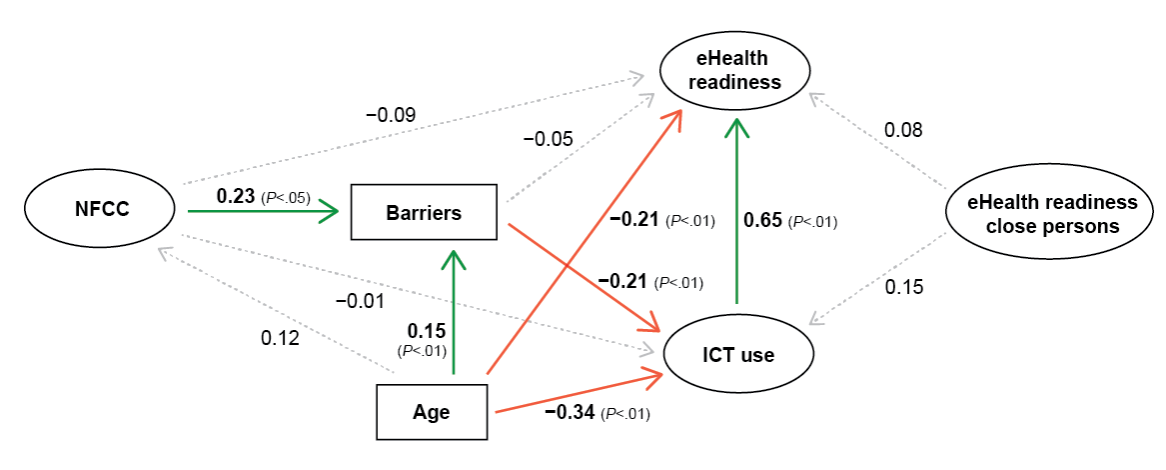
A simplified version of the tested model with path estimates. Ellipses indicate latent variables; rectangles indicate manifest variables; full colored arrows indicate significant relationships (green: positive; red: negative) whereas dashed arrows indicate nonsignificant relationships. eHealth: electronic health; ICT: information and communication technology; NFCC: need for cognitive closure.

On the basis of research questions and the hypothesized relationships, the structural model portrayed in Figure 1 was tested. With respect to the association between older adults’ and close persons’ eHealth readiness, the residual covariance between the items from the eHealth readiness scale (specifically item 1) was allowed based on residual correlation matrices, modification suggestions, as well as to account for the dyadic nature of the data and possible shared environment.

## Results

### Sample Characteristics

The mean age in the older adult sample was 66.14 (SD 9.47) years; 55.2% (138/250) were women. The majority of the older adults were retired (188/250, 75.2%) and completed high school education (171/250, 68.4%). In the sample of close persons, the mean age was 46.30 (SD 13.51) years; 70.4% (176/250) were women. Regarding the relationship toward the older adult, 54.8% (137/250) of close persons were children, 18.0% (45/250) were other relatives, and 15.2% (38/250) were partners, the rest accounting for friends, acquaintances, or professional caretakers. Detailed demographic characteristics of both samples can be found in Table 1.

The most widely used device among older adults was the cell phone, with 69.2% (173/250) of older adults using it on a daily basis. All the other devices were used by less than a fourth of the participants. Details on the mean daily usage (hours/day) of the ICT devices and internet among daily users as well as the total usage time and the total number of devices among all older adults can be found in Table 2. The table also provides details on the number of perceived barriers, NFCC, and eHealth readiness (for the older adults and their close persons as well).

Differences based on gender were examined and are portrayed in Table 2. In total, men used more ICT devices than women, and this difference was significant (t_248_=2.12 [2-talied *t* test]; *P*=.04; Cohen *d*=0.27). No other differences based on gender were significant.

As for the aggregated ICT usage variables, there were significant differences based on education in the total usage time (*F*_3,246_=3.23; *P*=.02), the number of used devices (*F*_3,246_=5.40; *P*=.001), as well as the number of perceived barriers (*F*_3,246_=3.72; *P*=.01). For usage time, college-educated participants reported higher time than participants with primary education, but the difference was not significant (mean difference 2.44; *P*=.05). Participants with primary education used fewer devices than participants with a high-school diploma (mean difference 0.43; *P*=.008) and to a lesser degree than participants with college education (mean difference 0.56; *P*=.005). Regarding the number of perceived barriers, the only significant group difference was between participants with primary education who perceived more barriers than college-educated participants (mean difference 0.87; *P*=.02). The most commonly perceived barriers by the older adults related to technologies evolving too fast (indicated by 96/250, 38.4% of older adults), fear of making a mistake (74/250, 29.6%), not being interested in internet technology (55/250, 22.0%), technologies being too expensive (48/250, 19.2%), and not having enough knowledge (47/250, 18.8%).

When comparing older adults with their close persons, close persons used on average more ICT devices (mean 2.03, SD 0.91; t_249_=−11.91; *P*<.001; Cohen *d*=0.958), used them for more hours per day (mean 5.32, SD 4.32; t_249_=−10.49; *P*<.001; Cohen *d*=0.76), and perceived fewer barriers to using ICT technology (mean 0.62, SD 0.95; t_249_*=*9.73; *P*<.001; Cohen *d*=0.79). Nevertheless, the sample of close persons was also significantly younger than the primary sample of older adults (t_249_= 22.55; *P*<.001; Cohen *d*=1.70).

**Table 1:** Demographic characteristics of older adults and their close persons.

Characteristic			Older adults		Close persons
**Age (years)**					
	Mean (SD)	66.14 (9.47)		46.30 (13.51)	
	Range	50-93		21-90	
**Gender, n (%)**					
	Females	138 (55.2)		176 (70.4)	
	Males	112 (44.8)		74 (29.6)	
**Education, n (%)**					
	Elementary	55 (22.0)		10 (4.0)	
	Secondary school (no diploma)	101 (40.4)		93 (37.2)	
	Secondary school (diploma)	70 (28.0)		108 (43.2)	
	University	24 (9.6)		39 (15.6)	
**Marital status, n (%)**					
	Married	88 (35.2)		133 (53.2)	
	Divorced	60 (24.0)		47 (18.8)	
	Widowed	89 (35.6)		9 (3.6)	
	Single	7 (2.8)		44 (17.6)	
	Living with a partner	6 (2.4)		17 (6.8)	
**Occupation, n (%)**					
	Employed	51 (20.4)		172 (68.8)	
	Retired	188 (75.2)		44 (17.6)	
	Unemployed	11 (4.4)		28 (11.2)	
	Students	N/A^a^		6 (2.4)	

^a^N/A: not applicable.

**Table 2:** Descriptive characteristics for the information and communication technology use variables, electronic health readiness, and need for cognitive closure.

Characteristic	Proportion of daily users (N=250), n (%)	Entire sample		Females		Males	
		Mean (SD)	Range	Mean (SD)	Range	Mean (SD)	Range
Cell phone usage (hours/day)	173 (69.2)	1.89 (3.31)	0.1-24.0	2.21 (4.22)	0.1-24.0	1.54 (1.82)	0.1-10.0
Smartphone usage (hours/day)	19 (7.6)	2.05 (1.92)	0.5-8.0	1.95 (1.61)	0.5-6.0	2.17 (2.32)	0.5-8.0
Personal computer usage (hours/day)	50 (20.0)	3.16 (2.52)	1.0-10.0	3.00 (2.49)	1.0-8.0	3.29 (2.60)	1.0-10.0
Laptop usage (hours/day)	39 (15.6)	2.64 (1.70)	0.1-7.0	2.49 (1.55)	0.1-6.0	2.81 (1.89)	0.5-7.0
Tablet usage (hours/day)	7 (2.8)	1.71 (0.76)	1.0-3.0	1.33 (0.58)	1.0-2.0	2.00 (0.82)	1.0-3.0
Internet usage (hours/day)	99 (39.6)	2.24 (1.98)	0.1-10.0	2.23 (1.92)	0.1-10.0	2.47 (2.02)	0.2-10.0
Total usage time (hours/day)	N/A^a^	2.43 (3.26)	0-16	2.26 (3.25)	0-16	2.64 (3.28)	0-14
Number of devices	N/A	1.24 (0.73)	0-4	1.15 (0.71)	0-4	1.35 (0.74)	0-4
Number of barriers	N/A	1.47 (1.20)	0-6	1.59 (1.25)	0-6	1.32 (1.13)	0-5
Need for cognitive closure	N/A	3.56 (0.56)	1.80-5.00	3.60 (0.54)	2.13-4.80	3.51 (0.59)	1.80-5.00
eHealth^b^ readiness	N/A	15.56 (7.55)	6-33	15.03 (7.49)	6-33	16.21 (7.60)	6-32
eHealth readiness close persons	N/A	22.76 (7.28)	6-36	23.07 (7.07)	6-36	22.38 (7.55)	6-35

^a^N/A: not applicable.

^b^eHealth: electronic health.

### Predicting Information and Communication Technology Use and eHealth Readiness

The evaluated structural model with standardized estimates of the regression paths is depicted in Figure 1. The model fit was good, χ^2^_438_=804.1; the χ^2^ to degree of freedom ratio was 1.84; CFI=0.911; TLI=0.899; SRMR=0.064; RMSEA=0.060; 95% CI (0.053-0.066), considering the complexity of the model, the sample size, and the initial fit of individual latent variables.

Estimates for the direct effects are displayed in Table 3. The results showed that the older an adult, the more barriers to using technology he/she perceived (β=.15; *P*=.008) and the lower his/her ICT use (β=−.34; *P*<.001) and eHealth readiness were (β=−.21; *P*<.001). Individuals with a high NFCC also perceived more barriers (β=.23; *P*=.01). Older adults who reported more barriers in fact used ICT less (β=−.21; *P*=.001). The relationship between ICT use and eHealth readiness of older adults was positive, meaning that individuals who use ICT more are also more prepared and willing to accept eHealth technology (β=.65; *P*<.001). None of the other direct effects were significant.

Indirect effect of NFCC on ICT use through perceived barriers was negative and significant (β=−.049; *P*=.04), meaning that a higher NFCC was related to more perceived barriers. Indirect effect of NFCC on eHealth readiness of older adults through perceived barriers and ICT use was negative and significant as well (β=−.031; *P*=.04). The expected direct effects of eHealth readiness of close persons on ICT use and eHealth readiness of older adults were not significant, but the total effect of eHealth readiness of close persons on eHealth readiness of older adults was positive and significant (β=.18; *P*=.02).

The evaluated model explained 63.7% of the variance of older adults’ eHealth readiness, 21.2% of variance of ICT use, and 8.5% of variance of the number of perceived barriers.

**Table 3:** Estimates of direct effects.

Regression path	Estimate B^a^	SE	*P* value	95% CI	Standardized estimate β
NFCC^b^ –> eHealth^c^ Readiness	−0.187	0.117	.11	−0.415 to 0.042	−.091
NFCC –> Barriers	0.750	0.301	.01	0.160 to 1.339	.232
NFCC –> ICT^d^ use	−0.055	0.574	.92	−1.180 to 1.070	−.008
Barriers –> eHealth Readiness	−0.034	0.028	.23	−0.090 to 0.022	−.053
Barriers –> ICT use	−0.429	0.133	.001	0.689 to −0.169	−.210
ICT use –> eHealth Readiness	0.201	0.034	<.001	0.134 to 0.268	.647
eHealth Readiness Close Persons –> eHealth Readiness	0.118	0.075	.12	−0.028 to 0.264	.080
eHealth Readiness Close Persons –> ICT use	0.730	0.432	.09	−0.117 to 1.577	.154
Age –> eHealth Readiness	−0.017	0.004	<.001	−0.025 to −0.008	−.207
Age –> Barriers	0.019	0.007	.008	0.005 to 0.034	.151
Age –> ICT use	−0.089	0.017	<.001	−0.122 to −0.056	−.342
Age –> NFCC	0.005	0.004	.24	−0.003 to 0.012	.117

^a^Nonstandardized estimate of direct effects (as compared with the standardized estimate β).

^b^NFCC: need for cognitive closure.

^c^eHealth: electronic health.

^d^ICT: information and communication technology.

## Discussion

### Principal Findings

This study evaluated the predictors of ICT use and readiness to use eHealth technology by older adults. The study considered the role of perceived barriers, the role of close persons’ eHealth readiness, and it was the first study to evaluate the influence of NFCC on ICT use and eHealth readiness of older adults. Although unable to definitively establish the direction of the studied relationships because of the cross-sectional nature of the data, the SEM analysis showed that apart from age, eHealth readiness was predicted by ICT use. Older adults who used ICT more in general were more ready to use technology for supporting health and healthy behaviors. Nevertheless, a reverse (higher eHealth readiness predicts more ICT use) or bidirectional relationship could also exist, but these were not specified and tested in the current model. Although the NFCC did not directly impact ICT use or eHealth readiness, NFCC exerted influence on ICT use and eHealth readiness indirectly through the number of perceived barriers. Individuals high in NFCC perceived more barriers to ICT. The number of barriers was, in turn, negatively related to their overall ICT use. Interestingly, there was evidence that significant others might influence older adults’ eHealth readiness, although the mechanisms remain unclear (the direct effect of close persons’ eHealth readiness on ICT use and eHealth readiness of older adults was not significant, but the total effect of close persons’ eHealth readiness on the older adults’ eHealth readiness was significant).

This study supported previous research [16-19] by finding that age plays an important role in ICT use and adoption. Older adults used ICT devices less, were less prepared to use it for monitoring and improving health, and perceived more barriers to ICT use than their younger counterparts. Similarly, in line with previous research [17-20], ICT usage was related to the level of education. Differences were found especially between older adults with primary education who used ICT to a lesser degree and perceived more barriers than college-educated older adults.

A novel predictor yielding interesting results was the NFCC. This research built upon the pioneering studies by Chernikova et al [35], which found NFCC to be related to intentions to use technological innovations and support the notion that NFCC may in fact play a role in technology adoption and use. The indirect effect of NFCC on ICT use and eHealth readiness through the number of perceived barriers (and in case of eHealth readiness through ICT use as well) suggests that individuals high in NFCC may perceive more barriers to ICT adoption and use, resulting in lower ICT use and eHealth readiness, but as a function of their reluctance to change and preference for tradition and security rather than the actual inability to use or learn to use ICT [34,51]. This notion should be evaluated in future research and should include a measure of perceived barriers by older adults as well as, for instance, a close person’s evaluation of the older adult’s actual barriers to technology adoption and use. Interestingly, NFCC indirectly predicted ICT use and eHealth readiness even when the effects of age were accounted for in the model, suggesting that this dispositional characteristic may, in part, help explain some of the age-related decline observed in ICT use.

The obtained results on the role of NFCC and perceived barriers on ICT use and readiness suggest interesting actionable implications. Researchers carrying out ICT use promoting interventions and programs might want to measure the level of NFCC of the participants in their programs. NFCC may then serve as a potential tailoring variable in the design of programs promoting ICT use, wherein depending on the individual’s level of NFCC, intervention components explicitly focusing on reducing the number of perceived barriers could be incorporated into the intervention. The respective strategies to mitigate barriers would depend on the specific barriers but could range from providing training with ICT devices to financial support to presenting older adults with available technology and how it can enhance one’s life. Effectively reducing perceived barriers (especially in high NFCC individuals) may then positively impact ICT acceptance and use. Moreover, NFCC has also been shown to be a situationally evocable state. This research suggests that when presenting a new ICT or eHealth device, application, or intervention to its potential users, it may be beneficial to lower their NFCC before and during the description of the device or intervention. This could be done, for example, by providing potential users with sufficient time when making decisions, by stressing the importance of forming an accurate judgment, or by aiding in the process of finding additional information on the matter before forming a judgement [33,52]. Experimental research testing these propositions is needed.

As for the role of significant others on older adults’ adoption and use of technology, which has been proposed by several theoretical approaches [17,53], the results are inconclusive at this point, and further research is needed to clarify this relationship. On the basis of these results, close persons might influence older adults’ eHealth readiness, but the mechanisms are not clear. The amount, level, and specifics of support of close persons to older adults regarding ICT and specifically eHealth technologies were not explicitly assessed in the present research. Further research could focus on evaluating the specific mechanisms of close persons’ influence on older adults’ ICT acceptance and use. A better understanding of the role that close persons play in the adoption of ICT technologies by older adults would be particularly useful when designing ICT use promotion interventions for older adults. Close persons could help facilitate ICT adoption by the provision of specific types of support or through other mechanisms yet to be identified.

### Limitations and Further Research

Considering the limited amount of research that has been conducted specifically on the relationship between NFCC and technology, this study should be regarded as an exploratory study, and the model should be confirmed and cross-validated on other samples to increase the validity of the results. A limitation of the present research is also the correlational nature of data, which does not allow for firm conclusions about the causality and direction of the relationships. Although the proposed relationships were theoretically construed and the proposed direction of causality seems theoretically plausible, further research should validate the findings in different older adult samples.

The sample size was rather low, considering the complexity of the evaluated models. It is also possible that an equally well-fitting structural model would result from a specification of different relationships. This further underscores the need for cross-validation and empirical testing of competing models in independent samples of older adults. Ideally, this research should involve studies with prospective, longitudinal, or experimental designs to allow for more definitive conclusions regarding the causality and time ordering of relationships under study.

Finally, little research has been conducted on the topic of ICT acceptance and use by Czech older adults—to our knowledge, we present the first systematic examination of ICT use and eHealth readiness in Czech older adults. Considering the rather low ICT usage and eHealth readiness in the current sample, it would be interesting to repeat the survey in a few years’ time to evaluate the changes. Similarly, comparing specific subsamples of the older adult population such as older adults who are physically active, older adults visiting university classes, and people living in homes for the elderly could yield information about further factors influencing ICT acceptance and use, helping shape ICT use promotion efforts and policies related to ICT adoption across the population spectrum of older adults.

### Conclusions

This is the first study to systematically evaluate Czech older adults’ readiness to use technology for improving health, their related ICT use, and possible predictors, including NFCC and the role of significant others. Our results provide new insights into the predictors of older adults’ readiness to use eHealth technology, especially with respect to NFCC. eHealth readiness was found to be affected directly by age and actual ICT use and indirectly by the NFCC and the number of perceived barriers toward using technology. These results are directly applicable for researchers or organizations carrying out interventions promoting the use of eHealth devices and applications.

Future researchers are encouraged to validate the findings in various older adult samples and further clarify the role of older adults’ close persons. Additional studies, including prospective or experimental studies, are required to support the presented findings.

## Abbreviations

CFI: comparative fit index
eHealth: electronic health
EU: European Union
ICT: information and communication technology
mHealth: mobile health
NFCC: need for cognitive closure
RMSEA: root mean square error of approximation
SEM: structural equation modeling
SRMR: standardized root mean square residual
TLI: Tucker-Lewis Index
